# Amygdala activity related to perceived social support

**DOI:** 10.1038/s41598-020-59758-x

**Published:** 2020-02-19

**Authors:** Wataru Sato, Takanori Kochiyama, Shota Uono, Reiko Sawada, Sakiko Yoshikawa

**Affiliations:** 1 Kokoro Research Center, Kyoto University, Kyoto University, 46 Shimoadachi, Sakyo, Kyoto, 606-8501 Japan; 2 Brain Activity Imaging Center, ATR-Promotions, 2-2-2 Hikaridai, Seika-cho, Soraku-gun, Kyoto, 619-0288 Japan; 3 Department of Neurodevelopmental Psychiatry, Habilitation and Rehabilitation, Graduate School of Medicine, Kyoto University, 53 Shogoin-Kawaharacho, Sakyo, Kyoto, 606-8507 Japan; 4 Faculty of Human Health Science, Graduate School of Medicine, Kyoto University, 53 Shogoin-Kawaharacho, Sakyo-ku, Kyoto, 606-8507 Japan

**Keywords:** Amygdala, Empathy

## Abstract

Perceived social support enhances well-being and prevents stress-related ill-being. A recent structural neuroimaging study reported that the amygdala volume is positively associated with perceived social support. However, it remains unknown how neural activity in this region and functional connectivity (FC) between this and other regions are related to perceived social support. To investigate these issues, resting-state functional magnetic resonance imaging was performed to analyze the fractional amplitude of low-frequency fluctuation (fALFF). Perceived social support was evaluated using the Multidimensional Scale of Perceived Social Support (MSPSS). Lower fALFF values in the bilateral amygdalae were associated with higher MSPSS scores. Additionally, stronger FC between the left amygdala and right orbitofrontal cortex and between the left amygdala and bilateral precuneus were associated with higher MSPSS scores. The present findings suggest that reduced amygdala activity and heightened connectivity between the amygdala and other regions underlie perceived social support and its positive functions.

## Introduction

The subjective perception of social support (i.e., the feeling of being supported by other people) plays a crucial role in human resilience and well-being. A number of psychological studies have shown that perceived social support prevents ill-being, such as perceived stress, anxiety, and depression^1–5^ and enhances well-being, such as subjective happiness^6–9^. To investigate this important psychological construct, psychometric studies have developed several questionnaires, including the Multidimensional Scale of Perceived Social Support (MSPSS^10^), that reliably and validly measure perceived social support. Subjectively perceived social support is related to received social support, but at only moderate strength, whereas it is more consistently linked to the aforementioned positive effects^11,12^. Based on these findings, it has been proposed that perceived social support is a stable characteristic, similar to a trait or a personality feature^11,13^.

To explore the neural mechanisms underlying perceived social support, a recent structural neuroimaging study has investigated the regional brain volumes associated with perceived social support as measured using the MSPSS^14^. The study reported that left amygdala volume is positively associated with MSPSS scores, which indicates that the amygdala plays an important role in the implementation of perceived social support.

However, the type of amygdala activity (i.e., hyper- or hypoactivity) related to perceived social support remains unknown. This information would be valuable for understanding the functional role of the amygdala in perceived social support. Several functional magnetic resonance imaging (fMRI) studies have indicated that low-frequency (<0.1 Hz) resting-state neural activity in the absence of a task is a suitable measure for assessing brain activity related to stable psychological constructs, such as perceived social support^15,16^. It has been proposed that the fractional amplitude of low-frequency fluctuation (fALFF) during the resting state reflects the intensity of spontaneous neural activity^17,18^. To our knowledge, no previous studies investigated the association between resting-state activity in the amygdala and perceived social support. Although one previous study measured resting-state brain activity and its association with MSPSS scores, the researchers only analyzed activity in predefined regions, such as the default mode network, and did not analyze amygdala-related activity^19^. However, findings from other literatures may provide some insight. Multimodal structural and functional MRI studies have consistently reported that lower amygdala volume is associated with higher amygdala activation during stressful or emotional tasks^20,21^. A positron emission tomography study also observed a positive association between resting-state amygdala activity and perceived stress scores^22^. Additionally, it was reported that participants with high anxiety and depression exhibit a lower volume and heightened resting-state activity in the amygdala^23,24^. Based on these findings, and in conjunction with ample evidence showing that people with high perceived social support generally have low levels of perceived stress or psychiatric problems^4^, we hypothesized that perceived social support would be negatively associated with fALFF values in the amygdala.

Furthermore, the characteristics of functional coupling between the amygdala and other brain regions associated with perceived social support remain unknown. Several previous fMRI studies have shown that resting-state functional connectivity (rsFC), or the correlations among spontaneous slow fluctuations of the hemodynamic signals of brain regions, are indicative of a functional neural system^15,25,26^. Of the various brain regions that exhibit rsFC with the amygdala^27^, several previous fMRI studies reported that rsFC between the amygdala and orbitofrontal cortex^28,29^ and between the amygdala and precuneus^30–32^ is reduced in depressed patients. Furthermore, rsFC between the amygdala and orbitofrontal cortex^33^ and between the amygdala and posterior cingulate gyrus^34^, which is adjacent to and densely connected with the precuneus^35^, was reduced following acutely stressful situations. A recent fMRI study also found that rsFC between the amygdala and precuneus is positively associated with subjective happiness^36^. Based on these data, in conjunction with psychological evidence showing that perceived social support is negatively associated with perceived stress, anxiety, and depression^4^ and is positively associated with subjective happiness^9^, we hypothesized that perceived social support would be positively associated with rsFC between the amygdala orbitofrontal cortex and between the amygdala and precuneus.

To test these hypotheses, we first analyzed the relationship between MSPSS scores^10^ and fALFF values in the amygdala. To better understand this association, we additionally analyzed fALFF values in the amygdala, controlling for related psychological constructs, its relationship with amygdala structure, and similar associations in other brain regions. Then, we investigated associations between MSPSS scores and rsFC between the amygdala seed regions and the other brain regions.

## Results

### Psychological ratings

The mean (±*SD*) perceived social support score was 5.2 (±1.1), which was comparable to that found in a previous study^37^ (*t*-test, *p* > 0.10).

### Association of fALFF with MSPSS scores

**Figure 1:**
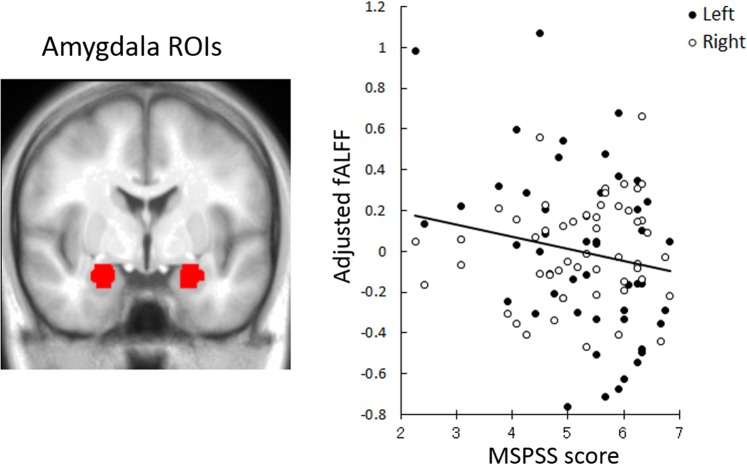
Significant negative associations between Multidimensional Scale of Perceived Social Support (MSPSS) scores and fractional amplitude of low-frequency fluctuation (fALFF) values of the amygdala. (**Left**) Regions of interest (ROIs) of the amygdala, which were derived from the Automated Anatomical Labeling atlas^38^. The area is overlaid on the mean spatially normalized structural magnetic resonance images. (**Right**) Scatter plots of the adjusted fALFF values of the left and right amygdalae as a function of MSPSS scores. Adjusted fALFF values were calculated by applying amygdala masks in the Automated Anatomical Labeling atlas, regressing out the effects of sex, age, full-scale intelligence quotient, and framewise displacement. Effects of no interest (hemisphere, hemisphere × MSPSS interaction, and constant term) were regressed out.

To investigate the relationship between MSPSS scores and amygdala activity, regions of interest (ROI) analyses were conducted. Mean fALFF values of each of the left and right amygdalae were calculated by applying amygdala masks in the Automated Anatomical Labeling (AAL) atlas^38^, adjusting for (regressing out) the effects of sex, age, full-scale intelligence quotient (IQ), and framewise displacement (FD)^39^. The adjusted fALFF values of the amygdala were then analyzed using a regression model with MSPSS scores, hemisphere, and their interaction as the independent variables. The results revealed a significant negative effect of MSPSS scores (*β* = −0.20, *t*(98) = 2.00, *p* < 0.05; Fig. 1) and a nonsignificant hemisphere effect or interaction (|*β*| < 0.13, *t*(98) < 1.36, *p* > 0.10), indicating that lower fALFF values in the bilateral amygdalae were associated with higher MSPSS scores.

We further conducted ROI analyses to test the association between MSPSS scores and adjusted fALFF values in the above model, controlling for related psychological constructs. We constructed regression models for the adjusted amygdala fALFF values with MSPSS scores, hemisphere, and their interaction as the independent variables and covariates including (1) the scores of five-factor personality domains (neuroticism, extraversion, openness to experience, agreeableness, and conscientiousness)^40^ for basic personality, (2) trait and state anxiety scores^41^ for general ill-being, and (3) subjective happiness scores^42^ for general well-being. The results confirmed significant negative relationships between MSPSS scores and fALFF values of the amygdala in all models (*β* < −0.22, *t* > 2.19, *p* < 0.05) and nonsignificant hemisphere effects or interactions (|*β*| < 0.14, *t*(98) < 1.39, *p* > 0.10). Among covariates, the positive effects of neuroticism, openness, and state anxiety were significant (*β* > 0.18, *t* > 1.72, *p* < 0.05).

Because we previously found that MSPSS scores are associated with global amygdala volume of the left amygdala^14^, we here investigated whether resting-state neural activity could be mediated by such a mind-structure relationship in the left amygdala. We conducted a mediation analysis^43^ using MSPSS scores and adjusted global volume values (regressing out the effects of sex, age, full-scale IQ, and total intracranial volume) and adjusted fALFF values of the left amygdala. The results showed that all steps were significant, including negative direct effects of MSPSS scores on fALFF values, positive indirect effects of MSPSS scores on global volume values, and negative indirect effects of global volume values on fALFF values (|*β*| > 0.28, *t* > 2.00, *p* < 0.05) (Supplementary Fig. 1). Furthermore, a significant mediation effect of global volume values on the relationship between MSPSS scores and fALFF values was observed (MacKinnon’s *z*′ test^44^, *z*′ = 1.67, *p* < 0.01).

To search for other regions in terms of the associations between MSPSS scores and fALFF values, a voxel-based whole brain analysis was conducted with an extent threshold of *p* < 0.05 and a family-wise error rate corrected with a cluster-forming threshold of *p* < 0.001 (uncorrected). This analysis showed no significant region. For descriptive purposes, we conducted analyses with a liberal threshold (extent threshold of uncorrected *p* < 0.05 with a cluster-forming threshold of uncorrected *p* < 0.001). The results showed associations between MSPSS scores and fALFF values in several brain regions, including a positive association in the left precuneus (Supplementary Table 1).

### rsFC associated with MSPSS scores

**Figure 2:**
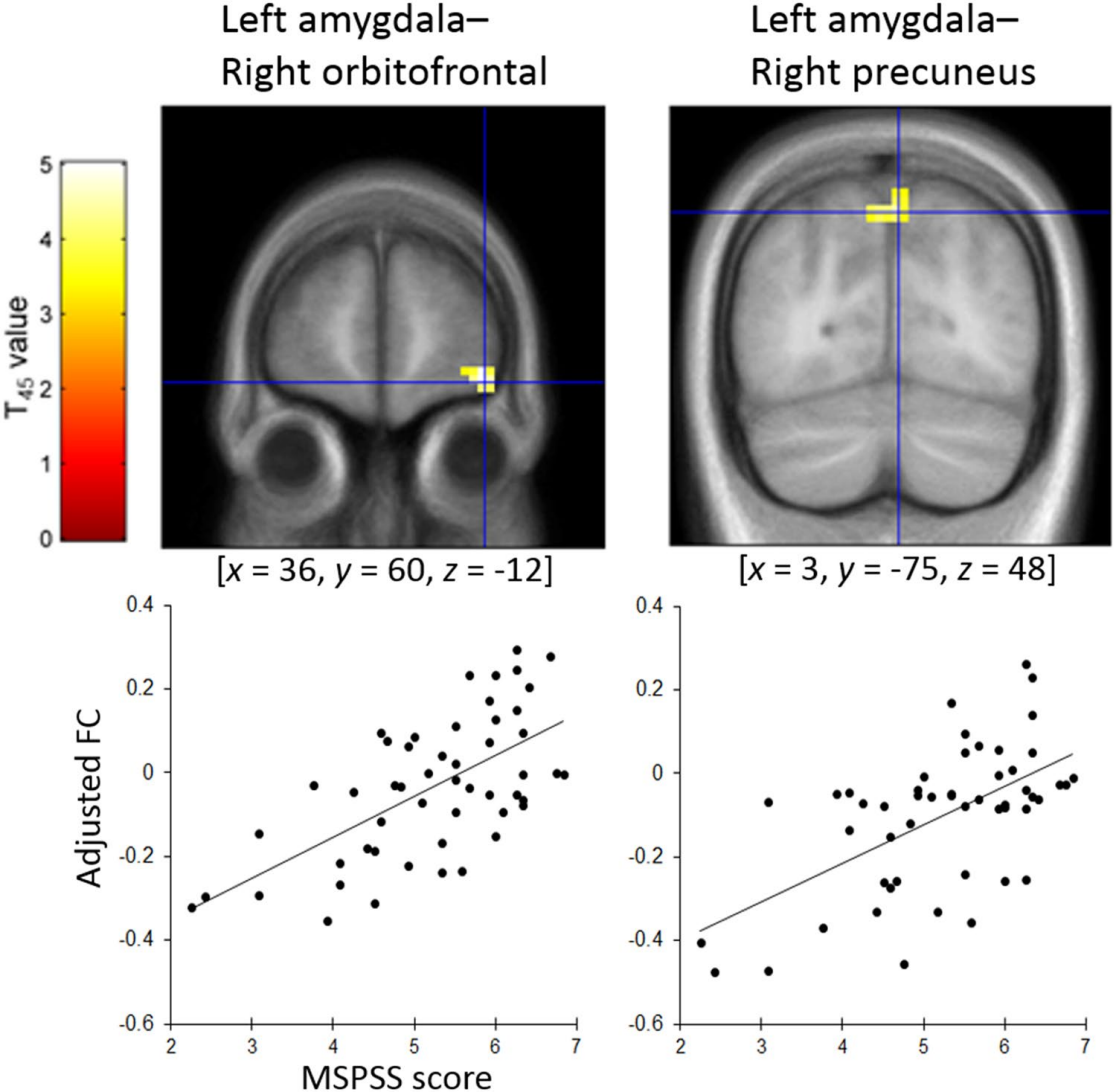
Brain regions showing a significant positive association between the Multidimensional Scale of Perceived Social Support (MSPSS) score and resting-state functional connectivity (rsFC) values seeded from the left amygdala. (**Upper**) Statistical parametric maps (*p* < 0.05, family wise error corrected for the whole brain) for the group analysis of the seed-based functional connectivity map. The area is overlaid on the mean spatially normalized structural magnetic resonance images. The blue cross indicates the location of the peak voxel and the red–yellow color scale indicates the *T*-values. (**Lower**) Scatter plots and regression lines of the adjusted rsFC parameters as a function of the MSPSS scores at the peak voxels. Effects of no interest (age, sex, full-scale intelligence quotient, and mean framewise displacement) were regressed out.

To identify spontaneous functional coupling associated with the MSPSS scores, left and right amygdala seed rsFC was assessed for the whole brain after sex, age, full-scale IQ, and FD were regressed out. MSPSS scores were significantly and positively associated with rsFC between the left amygdala and right orbitofrontal cortex (*x* = 36, *y* = 60, *z* = −12; *T*(45) = 5.12; 891 mm^3^; Brodmann’s area (BA) 11) and between the left amygdala and bilateral precuneus (*x* = 3, *y* = −75, *z* = 48; *T*(45) = 4.49; 945 mm^3^; BA 7) (Fig. 2). A marginally significant positive association between MSPSS scores and rsFC was also observed between the right amygdala and bilateral precuneus (*x* = 6, *y* = −60, *z* = 51; *T*(45) = 4.12; 648 mm^3^; BA 7; *p* < 0.10, cluster-level corrected). No other regions showed significant rsFC with left or right amygdala activity associated with MSPSS scores.

## Discussion

Analysis of the fALFF values revealed a negative association between MSPSS scores and resting-state neural activity in the left and right amygdalae. This association was not accounted for by related psychological constructs, including basic personality, general well-being, and general ill-being. The involvement of amygdala activity in perceived social support is consistent with the results of a previous structural MRI study^14^, although the type of activity was unknown. The amygdala involvement in perceived social support observed in the present study is also consistent with ample evidence from lesion and recording studies in humans and monkeys, showing that the amygdala plays an indispensable role in various types of social functions^45,46^. Extending these prior findings, the present study provides the first evidence that higher perceived social support is associated with decreased resting-state neural activity in the amygdala.

Our analyses with covariates of five-factor personality domains, trait and state anxiety, and subjective happiness confirmed the negative associations between MSPSS scores and the fALFF values of the amygdala. The results suggest that the association between perceived social support and resting-state amygdala activity could not be accounted for by these psychological constructs. The results revealed that MSPSS scores and state anxiety scores were independently associated with fALFF activity of the amygdala with opposite effects, positively and negatively, respectively. The positive association between anxiety and the amygdala is consistent with previous findings that heightened activity in the amygdala was associated with anxiety and depression^24,47,48^ as well as perceived stress level^22^. Collectively, these antagonistic relationships between perceived social support and ill-being on amygdala activity may underlie the well-validated relationship between perceived social support and ill-being, including perceived stress, anxiety, and depression^4^.

Mediation analysis revealed that relationships between MSPSS scores and fALFF values of the amygdala were mediated by global volume values of the amygdala. Specifically, a positive association was found between MSPSS scores and amygdala structure; negative associations between structural volume and resting-state activity of the amygdala and between MSPSS scores and resting-state amygdala activity were obtained. The negative relationship between structural volume and resting-state activity is in agreement with previous multimodal imaging studies showing a negative relationship between volume and task-based activity in the amygdala^20,21^. Negative relationships between the volume and resting-state activity have also been reported in other regions, such as the hippocampus^49^ and precuneus^50^, whereas some studies have identified positive relationships in certain regions^51^. Although how the neural structure and activity are associated remains largely unclear, these data suggest that the negative relationships between perceived social support and reduced resting-state activity in the amygdala is mediated by its volume.

Additionally, the present study observed stronger rsFC between the amygdala and orbitofrontal cortex and between the amygdala and precuneus that was associated with higher MSPSS scores. These results are consistent with evidence from monkeys showing anatomical connections between the amygdala and orbitofrontal cortex^52^ and between the amygdala and medial parietal region^35,53^. Because the amygdala–orbitofrontal and amygdala–precuneus rsFC values are negatively associated with anxiety^54^, depression^30^, and perceived stress^33^ and amygdala–precuneus rsFC is positively associated with subjective happiness^36^, rsFC may account for these psychological activities associated with perceived support^4^. Alternatively, but not mutually exclusively, rsFC between the amygdala and these regions may be involved in other functions associated with perceived social support. For example, neuroimaging and lesion studies have shown that the orbitofrontal cortex is involved in emotion regulation^55–57^, specifically via functional coupling with the amygdala^58,59^. Thus, rsFC between the amygdala and orbitofrontal cortex may be related to an enhanced ability for adaptive emotional regulation that is associated with perceived social support^1,60^. Similarly, because activity in the precuneus has been shown to be associated with self-referential mental activity^61–63^, rsFC between the amygdala and precuneus may be related to heightened self-esteem states depending on higher perceived social support^4,64^. Collectively, the present results suggest that the heightened rsFC between the amygdala and orbitofrontal cortex and between the amygdala and precuneus that were associated with higher perceived social support could be related to the positive effects of perceived social support.

Several limitations of this study should be acknowledged. First, the present study included a small sample, which may have resulted in a lack of power for detecting associations between perceived social support and other brain regions. Our analyses using a liberal threshold revealed positive associations between MSPSS scores and fALFF values in several brain regions, including the precuneus, which is consistent with the results of a previous study^19^. The data suggest that more brain regions may be involved in the production of perceived social support. Second, we did not assess objective social measures, such as received social support and number of social connections. Because previous studies have demonstrated that such objective social measures are positively (although moderately) correlated with perceived social support scores^11,12^ and are positively associated with the amygdala structure^65–68^, objective social situations may explain some of the present findings. Finally, our results did not specify specific psychological or biological processing, mediating perceived social support and amygdala activity. As candidates for such mediators, previous neuroimaging studies have shown that specific psychological activity (e.g., affiliative sentiments^69,70^ and decision making under uncertainty^71^), neurotransmitters (e.g., oxytocin^69,72^), and expression of genes (e.g., rs6265^73^) are related to amygdala activity and connectivity. Further studies testing a large sample and assessing objective social measures and possible mediator variables would deepen our understanding of the neurocognitive mechanisms underlying perceived social support.

In conclusion, the present study demonstrated that lower fALFF values in the bilateral amygdalae were associated with higher MSPSS scores. Additionally, stronger rsFC between the left amygdala and right orbitofrontal cortex and between the left amygdala and bilateral precuneus were associated with higher MSPSS scores. Together with other evidence of functions in these regions, the present findings suggest that reduced amygdala activity and heightened amygdala–orbitofrontal and amygdala–precuneus couplings underlie perceived social support and its positive functions.

## Methods

### Participants

Fifty-one Japanese volunteers (26 females; mean ± *SD* age, 22.5 ± 4.5 years) participated in this study. The sample size was determined according to a previous methodological study’s recommendation that approximately 45 participants are needed to achieve a statistical power of 0.80^74^ with a standard effect size (Cohen’s *d* = 1) using a voxel-wise whole-brain correction^75^. All participants participated in semi-structured interviews by a psychologist using the Mini-International Neuropsychiatric Interview^76^, and none showed any neuropsychiatric conditions. All participants completed the Edinburgh Handedness Inventory^77^ and showed right hand dominance. After receiving the explanation of procedures, all participants provided written informed consent. This study was approved by the Ethics Committee of the Primate Research Institute, Kyoto University, and was conducted according to institutional ethical provisions and the Declaration of Helsinki.

### Psychological questionnaires

We used the MSPSS Japanese version^10,37^ to assess perceived social support. This questionnaire has a total of 12 items that measure subjective social support (e.g., “I get the emotional help and support I need from my family”) from the sources of either family, friends, or significant others. Each of these sources consisted of four items. Each item on the scale ranged from very strongly disagree (1) to very strongly agree (7). The reliability and validity of the questionnaire for Japanese participants was confirmed in a previous study^37^.

We used other questionnaires to assess psychological constructs related to perceived social support. First, we assessed the participants’ five domains of personality to confirm that the results could not be accounted for by their basic personality. The NEO Five-Factor Inventory Japanese version was applied^40,78^. This questionnaire has a total of 60 items that measure the personality domains of neuroticism, extraversion, openness to experience, agreeableness, and conscientiousness. Each of these domains consisted of 12 items. Second, we assessed the participants’ state and trait anxiety level using the State-Trait Anxiety Inventory Japanese version^41,79^. This questionnaire has a total of 40 items that measure state or trait anxiety level with 20 items for each. Third, we assessed the participants’ subjective happiness using the Subjective Happiness Scale Japanese version^42,80^. This questionnaire has a total of four items that measure global subjective happiness. The reliability and validity of these questionnaires with Japanese participants has been verified in previous studies^78–80^.

We also measured participants’ IQs, because the subjective ratings can be confounded by intelligence^81^. We used the revised Wechsler Adult Intelligence Scale, third edition (Nihon Bunka Kagakusha, Tokyo, Japan). The present study was part of a larger project that investigated mental health.

### Procedure

The participants engaged in a resting-state task for 5 min. The participants viewed a display that was projected from a liquid crystal projector (DLA-HD10K; Japan Victor Company, Yokohama, Japan) to a mirror positioned in front of them. On the display, a white fixation point (a small “+”) was continuously presented at the center of a black background using Presentation 16.0 software (Neurobehavioral Systems, Albany, CA, USA). The participants were requested to keep their eyes open, look at the fixation point, and relax without considering any specific content.

### MRI acquisition

All images were acquired using a 3-T scanning system (MAGNETOM Trio, A Tim System, Siemens, Malvern, PA, USA) with a 12-channel head coil at the ATR Brain Activity Imaging Center. Elastic pads were set on both sides of the head to minimize motion artifacts. Functional images were obtained using a T2*-weighted gradient-echo echo-planar imaging sequence. Image volume consisted of 39 consecutive slices parallel to the plane of the anterior–posterior commissure in ascending order, covering the whole brain. The imaging parameters were as follows: repetition time (TR) = 2,500 ms; echo time (TE) = 30 ms; flip angle = 80°; matrix size = 64 × 64; and voxel size = 3 × 3 × 4 mm^3^. Following functional image acquisition, a T1-weighted high-resolution structural image was acquired using a magnetization-prepared rapid-acquisition gradient-echo sequence. The parameters were: TR = 2,250 ms; TE = 3.06 ms; flip angle = 9°; inversion time = 1,000; GRAPPA acceleration factor = 2; 208 sagittal slices; slice thickness = 1 mm; field of view = 256 × 256 mm^2^; and voxel size = 1 × 1 × 1 mm^3^.

### Image analysis

Image analyses were conducted with the Statistical Parametric Mapping 12 package (http://www.fil.ion.ucl.ac.uk/spm) and Data Processing Assistant for Resting-State fMRI V4.0^81^ in the Data Processing & Analysis for Brain Imaging V2.0 (http://rfmri.org/dpabi)^82^ implemented in MATLAB R2017b (MathWorks; Natick, MA, USA). Major steps included preprocessing, fALFF calculation, rsFC calculation, and statistical analysis.

#### Preprocessing

The initial five volumes were deleted, and the remaining 120 volumes were preprocessed. Functional images were corrected to account for differences in slice timing and head movements. None of the participants exhibited large (>2 mm) motion corrections. To check motion artifacts, FD, defined as the sum of the absolute derivative values of the six realignment parameters^39^ was calculated. Data from all participants showed small FD (<0.25; mean ± *SD*, 0.11 ± 0.04). The FD values were further used for calculating nuisance regressors and effect-of-no-interest covariates at the individual and group level analyses, respectively.

A nuisance covariate regression was then conducted in native space before the spatial normalization to minimize the effects of noise (e.g., cardiac and respiratory cycles). In the general linear model of the fMRI time series, the effects of nuisance covariates, including six realignment parameters, one prior time point of the six realignment parameters, the 12 corresponding squared items (i.e., Friston 24-parameter model^84^), the linear trend, the white matter and cerebrospinal fluid signals, the mean global signal, and a constant term were regressed out. For the rsFC analysis, spike regression^83^ was used to correct for motion-contaminated volumes identified using a threshold of FD > 0.5 mm as well as one neighbor back and two neighbors forward. In the general linear model, a separate nuisance regressor had values of 1 and 0 at the contaminated time point and elsewhere, respectively. In previous studies, the combination of these nuisance regressors were shown to effectively remove motion-related artifacts^83,85,86^.

After the T1 images were coregistered onto the mean of functional images, the T1- and noise-corrected functional images were normalized into the Montreal Neurological Institute space using the anatomical image-based unified segmentation–spatial normalization approach^87^. Finally, the noise-corrected and spatially normalized functional images were resampled to 3 × 3 × 3 mm^3^ and smoothed with 4-mm full-width at half-maximum Gaussian filter, which is recommended for analyses of fALFF and rsFC^88^.

#### fALFF calculation

fALFF was computed using the individual preprocessed functional images^17,18^. fALFF is the ratio between the averaged square root power spectrum within a specific low-frequency range (0.01–0.1 Hz) and within the entire frequency range (0–0.2 Hz). The fALFF value for each voxel was calculated, and then *z*-standardized fALFF value to reduce potential variability of the global effects across participants. The resultant standardized fALFF values were entered into the group level analysis.

#### rsFC calculation

A seed-based rsFC was calculated to measure rsFC between the amygdala and other brain regions. First, the individual preprocessed functional images were bandpass-filtered at 0.01–0.1 Hz and the bilateral amygdala ROIs were obtained using the AAL atlas^38^. Then, time series data were extracted from each amygdala seed in the filtered data and Pearson’s correlation coefficients were calculated between the time series of the amygdala and all other voxels in the brain. The correlation coefficient at each voxel was *z*-transformed using Fisher’s *r*-to-*z* transformation to satisfy the normality assumption. The resultant amygdala rsFC maps were entered into the group level analysis.

#### Statistical analysis

To determine the association between MSPSS scores and fALFF values, we first conducted an ROI analysis for the amygdala based on predictions. The fALFF values of the left and right amygdalae were extracted from each participant using the first eigenvariate of all voxels within amygdala masks in the AAL atlas^38^ and adjusting for (regressing out) the effects of sex, age, and full-scale IQ, as well as the mean FD, which was an average across the entire time series, to minimize the impact of motion-related variance on the group inference^39,83^. The adjusted fALFF values of the bilateral amygdalae were used as the dependent variable in a multiple regression analysis using a regression model with MSPSS scores, hemisphere, and their interaction as the independent variables. The effect of MSPSS was tested using a *t*-test (one-tailed).

ROI analyses for adjusted fALFF values of the amygdala were further conducted in regression models with covariates of relevant psychological constructs. We constructed the regression models based on the above analysis with (1) the scores of five-factor personality domains, (2) trait and state anxiety scores, or (3) subjective happiness scores as covariates. The effects of MSPSS were tested using *t*-tests (one-tailed). The covariates were also evaluated using *t*-tests (one-tailed).

ROI analyses were also conducted to test the mediation analysis^43^ using the Variational Bayesian Analysis toolbox (https://mbb-team.github.io/VBA-toolbox/)^89^. We tested the mediation model in which adjusted fALFF values of the left amygdala mediate the relationship between the adjusted values of global volume of the left amygdala and MSPSS scores. The adjusted values of global volume of the left amygdala were calculated using FIRST in the FMRIB Software Library (FSL) ver. 5^90^, with standard automated subcortical segmentation procedures^14,91^. The effects of sex, age, full-scale IQ, and the total cerebral volume were regressed out. The meditation effect was tested using MacKinnon’s *z*′ test^44^ (one-tailed), which was validated for statistical power to detect medium effect size with a sample size of 50 in a simulation study^44^; in contrast, the Sobel test^92^, which is the most commonly used, was shown to lack such statistical power.

We also conducted exploratory analyses of the relationship between MSPSS scores and fALFF values using a voxel-wise multiple regression analysis for the whole brain. The regression model was identical to the above ROI analysis. Clusters were considered statistically significant if they reached the extent threshold of *p* < 0.05 with a family-wise error rate corrected with a cluster-forming threshold of *p* < 0.001 (uncorrected) using random field theory^93^. For descriptive purposes, analyses were also conducted with the extent threshold of *k* > 10 with a cluster-forming threshold of *p* < 0.01 (uncorrected).

For the analysis of the association between MSPSS scores and rsFC of the bilateral amygdala seed regions, a voxel-wise multiple regression analysis was conducted with rsFC as the dependent variable and with the same independent variable and covariates, as well as the thresholds, as the above whole brain fALFF analyses.

The brain structures were anatomically labeled and identified according to BAs using the AAL atlas^38^ and Brodmann Maps (Brodmann.nii), respectively, using the MRIcron software (www.mccauslandcenter.sc.edu/crnl/mricron/).

## Supplementary information

Supplementary Table 1 and Supplementary Figure 1.
