# Supplementary material for: Amygdala activity related to perceived social support
Source: Sci Rep. 2020 Feb 19;10:2951. doi: 10.1038/s41598-020-59758-x (PMC7031379; doi:10.1038/s41598-020-59758-x)
Supplement: Supplementary file 1 — Supplementary Table 1 and Supplementary Figure 1. [file 41598_2020_59758_MOESM1_ESM.doc]

Amygdala activity related to perceived social support

Wataru Sato, Takanori Kochiyama, Shota Uono, Reiko Sawada, and Sakiko Yoshikawa

Supplementary data

Supplementary Table 1. Brain regions that exhibited significant associations between Multidimensional Scale of Perceived Social Support scores and fractional amplitude of low-frequency fluctuation values with a liberal threshold (extent threshold of uncorrected *p* < 0.05 with a cluster-forming threshold of uncorrected *p* < 0.001).

| Brain region | BA |  | Coordinates | | | *Z*-value | Cluster Size |
| --- | --- | --- | --- | --- | --- | --- | --- |
|  |  |  | x | y | z |  | (voxels) |
| **Positive** |  |  |  |  |  |  |  |
| L. middle frontal gyrus | 6 |  | -24 | 0 | 45 | 3.99 | 3 |
| L. superior occipital gyrus | 18 |  | -15 | -90 | 33 | 3.88 | 5 |
| L. supramarginal gyrus | 2 |  | -57 | -33 | 30 | 3.74 | 3 |
| R. superior parietal lobule | 7 |  | 27 | -63 | 69 | 3.49 | 4 |
| L. precuneus | 7 |  | -27 | -54 | 54 | 3.48 | 3 |
|  |  |  |  |  |  |  |  |
| **Negative** |  |  |  |  |  |  |  |
| R. inferior temporal gyrus | 37 |  | 45 | -48 | -9 | 4.09 | 3 |
| R. precental gyrus | 6 |  | 30 | -3 | 60 | 3.74 | 4 |
| R. cuneus | 19 |  | 12 | -78 | 36 | 3.67 | 4 |
| R. Rolandic operculum | 48 |  | 45 | -9 | 15 | 3.35 | 3 |
| L. amygdala | - |  | -33 | -3 | -21 | 3.36 | 4 |

BA, Brodmann’s area; L, left; R, right.


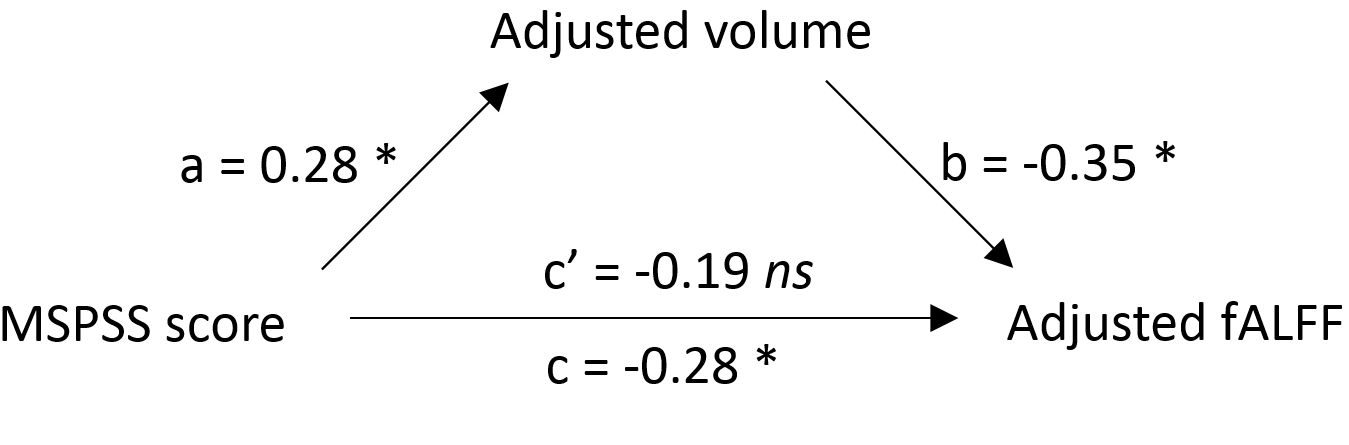


Supplementary Figure 1. Mediation analysis results for Multidimensional Scale of Perceived Social Support (MSPSS) scores and global volume values and adjusted values of fractional amplitude of low-frequency fluctuation (fALFF) of the left amygdala. Standardized coefficients are shown. *, *p* < 0.05; *ns*, non-significant.
